# *Candida albicans* Virulence Factors and Pathogenicity for Endodontic Infections

**DOI:** 10.3390/microorganisms8091300

**Published:** 2020-08-26

**Authors:** Yeon-Jee Yoo, A Reum Kim, Hiran Perinpanayagam, Seung Hyun Han, Kee-Yeon Kum

**Affiliations:** 1 Department of Comprehensive Treatment Center, Seoul National University Dental Hospital, Seoul 03080, Korea; duswl32@snu.ac.kr; 2 Department of Oral Microbiology and Immunology, School of Dentistry, Dental Research Institute and BK21 Plus Program, Seoul National University, Seoul 08826, Korea; kimareum@snu.ac.kr (A.R.K.); shhan-mi@snu.ac.kr (S.H.H.); 3 Schulich School of Medicine & Dentistry, University of Western Ontario, London, ON N6A 5C1, Canada; hperinpa@uwo.ca; 4 Department of Conservative Dentistry, Dental Research Institute and BK21 Plus Program, Seoul National University Dental Hospital, Seoul National University School of Dentistry, Seoul 03080, Korea; 5 National Dental Care Center for Persons with Special Needs, Seoul National University Dental Hospital for Persons with Special Needs, Seoul 03080, Korea

**Keywords:** biofilm, *Candida albicans*, endodontics, intraradicular eradication, pathogenicity, persistent infection, virulence factors

## Abstract

*Candida albicans* (*C. albicans*) is the fungus most frequently isolated from endodontic root canal infections. Although recognized by dental pulp and periradicular tissue cells that elicit immune responses, it eludes host defenses and elicits cell death. Then, *C. albicans* binds tooth dentin, forms biofilms, and invades dentinal tubules to resist intracanal disinfectants and endodontic treatments. Insensitive to most common medicaments, it survives sequestered within biofilms and intratubular dentin. Thus, *C. albicans* has been associated with cases of persistent or refractory root canal infections. Its treatment strategies may require alternative intracanal irrigants, intracanal medicaments such as chlorhexidine gel or human beta defensin-3 (HBD3), Ca-Si-based obturating materials, and microsurgical procedures.

## 1. *Candida* Infection in Endodontics

Microbial infections of the dental pulp and root canals can lead to an inflammatory disease in the periradicular tissues, known as apical periodontitis. These endodontic infections develop through pulp exposures due to dental caries, traumatic tooth fractures, or cracks [1]. Fungi are frequently involved and have been isolated from approximately 3–18% of infected root canals [2], with a predominance of the *Candida* species [2,3]. A systematic review and meta-analysis showed that *Candida albicans* is the fungus most commonly isolated from infected root canals, followed by *Candida tropicalis*, *Candida kefyr*, *Candida parapsilosis*, *Candida glabrata*, *Candida krusei*, *Candida dubliniensis*, *Candida guilliermondii*, and *Candida etchellsii* [3].

**Figure 1:**
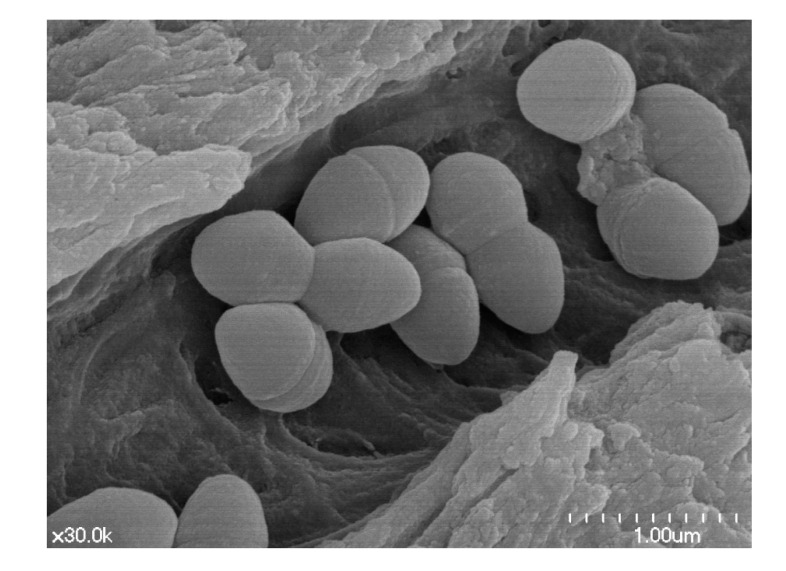
Scanning electron microscopic view of *Candida albicans* cells penetration into dentinal tubules after 3-weeks infection in human root dentin block. Blastospores and cells showing germ tube formation (30,000×).

*C. albicans* binds to both biotic and abiotic surfaces, such as dental prostheses and tooth dentin. It is dentinophilic and colonize the dentin walls of root canals, penetrating the dentinal tubules and forming biofilms (Figure 1). The round *C. albicans* cells attach to dentin surfaces over 60–90 min [4,5,6], and then proliferate to form a basal layer of biofilm that matures in 24 h. Mature biofilms contain multiple layers of polymorphic cells consisting of hyphal, pseudohyphal, and yeast forms that are embedded within extracellular matrices, creating thick and physiochemically hard structures. Then, round yeast cells from mature biofilms disperse to infect distant sites. *C. albicans* within biofilms is 10–100 fold more resistant to host immune responses and antifungal treatment, because the cell growth and metabolism are slowed and protected by extracellular polymeric substances (EPS) and protective factors [5,6]. Thus, *C. albicans* in biofilms is more difficult to remove than planktonic cells, and it is commonly found in persistent/refractory endodontic infections that do not respond to root canal treatment.

*C. albicans* is dimorphic and switches from round yeast cells to hyphal forms that invade and induce host immune responses [7]. Yeasts bind oral epithelial cells and rapidly switch to hyphae [8,9] that invade via two mechanisms [7,9]. Adhesins on the cell wall of hyphae induce endocytosis in the host cells [10,11]. These agglutinin-like sequence (ALS) proteins on hyphae promote host cell adherence and endocytosis [12]. For example, ALS3 regulated by Bcr1 is a C_2_H_2_ zinc finger protein of *C. albicans* that attaches to host receptors including N- and E-cadherins, and induces endocytosis in the host cells [11,13,14]. Additionally, hyphae can actively penetrate the plasma membrane of epithelial cells [7]. Both mechanisms of invasion occur in oral epithelium, whereas only active penetration occurs in intestinal epithelial cells.

**Figure 2:**
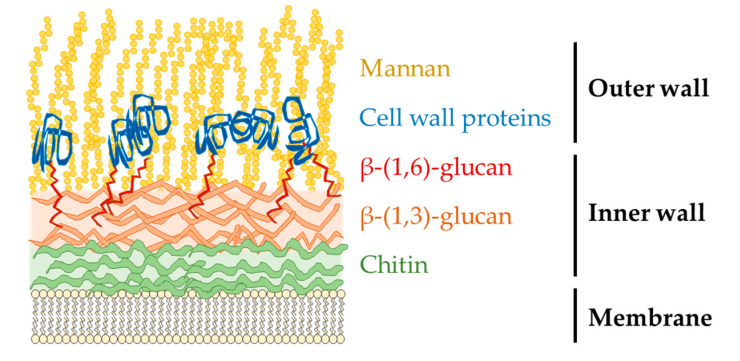
*Candida albicans* cell wall structures in yeast and hyphal forms have similar composition. However, chitin in the inner wall of hyphae is at least three times thicker than in yeast, and the amount of mannose in the mannan of yeast is higher than in that of hyphae [19,20].

The adherence to surfaces is mediated through a thick cell wall, with a core structure consisting of chitin, β-(1,3)-glucan, and β-(1,6)-glucan (Figure 2). β-(1,3)-glucan is covalently linked to other components forming a three-dimensional network [15,16]. Adjacent to this core complex lies the membrane as an inner layer with components that can be exposed to host cells during yeast budding [17]. The other end is the outer layer consisting of mannan and various cell wall proteins [16], which are linked to inner layer components primarily though glycosylphosphatidylinositol to β-(1,3)-glucan or β-(1,6)-glucan to chitin. The cell wall proteins are highly glycosylated with mannan, with *O*-linked mannan through their Ser/Thr-rich domains, and *N-*linked mannan with a conserved (Mannose)_8_(*N*-acetylglucosamine)_2_ structure as a core attached to diverse outer chains. The outer chain consists of an α-(1,6)-mannose backbone, and α-(1,2)-β-(1,2)- and α-(1,3)-linked mannose and phosphomannose as side chains [15,16,18]. The exposure of these components can induce host immune responses.

## 2. Virulence Factors and the Pathogenetic Mechanism in Pulp and Periapical Lesions 

As root canals are colonized and invaded by *C. albicans* [3,21], it encounters host cells and elicits an immune response from periapical tissues. Histopathologically, chronic apical periodontitis lesions involve periapical granulomas and sometimes apical cysts. Granulomas are chronic inflammatory tissues heavily infiltrated with neutrophils, macrophages, T- and B-lymphocytes, mast cells, osteoclasts, osteoblasts, fibroblasts, and epithelial cells, and they are enclosed by a fibrous capsule [22,23,24,25,26]. A periapical cyst consists of an epithelial-lined cavity within the granuloma [22,23,24].

### 2.1. Recognition of C. albicans by Immune Cells 

Cell wall components such as chitin, glucan, or mannans, DNA, RNA, and quorum-sensing molecules in *C. albicans*, are involved in recognition by monocytes and macrophages (Table 1). These involve host pattern recognition receptors (PRRs), such as Toll-like receptors (TLR), c-type lectin receptor (CLR), nucleotide-binding oligomerization domain-like receptors (NLR), and retinoic-acid inducible gene-1-like receptors (RLR) [27].

Phospholipomannan on *C. albicans* is recognized by TLR2 on macrophages that activate nuclear factor-κB (NF-κB) and produce tumor necrosis factor (TNF)-α. [28]. Mannan purified from *C. albicans* induced TNF and Interleukin 6 (IL-6) in human mononuclear cells and murine macrophages, whereas mannosylation-defective mutants stimulated significantly less of both cytokines. *O-* and *N*-linked mannosyl residues induced cytokines via TLR4 and mannose receptors, respectively [29]. Mannan from *C. albicans*, and not *Saccharomyces cerevisiae*, induced IL-17 in human peripheral blood mononuclear cells (PBMCs), which was dependent on macrophage mannose receptors [30]. Mannose receptor-mediated IL-17 release is enhanced by the TLR2/dectin-1 pathway. Both TLR2 and dectin-1 receptors recognized β-glucan in the cell wall of *C. albicans* and induced cytokine production [29]. Thus, mannan and β-glucan may together induce more severe inflammatory responses.

β-glucans bind to dectin-1, an extracellular CLR, which stimulates macrophages and dendritic cells to phagocytose *C. albicans*. Dectin-1 recognizes *C. albicans* in their yeast form, but not as hyphae or pseudohyphae. β-glucans are unexposed during hyphal or pseudohyphal growth, but are exposed during yeast budding and cell division to trigger phagocytosis and the production of reactive oxygen [17]. This non-exposure of β-glucans in hyphae may be a strategy for *C. albicans* to evade phagocytosis by macrophages. Other CLRs that recognize *C. albicans* include Mincle, which has a key role in the immune response against *C. albicans* [31]. *C. albicans* induced TNF-α production in macrophages, which was reduced in Mincle-deficient mice that were more susceptible to the infection.

On the other hand, a soluble CLR, mannan-binding lectin (MBL), inhibited *C. albicans*-induced proinflammatory cytokines and chemokine production in human macrophages by inhibiting the *C. albicans*-mediated TLR2 and TLR4 expression and blocking TLR2 and TLR4 activation [32]. However, multiple CLRs, including Dectin-1, Dectin-2, and Mincle, collaborate to remove *C. albicans* during systemic infections [33]. The protective effect of multiple CLRs over single receptors was potently enhanced in monocytes, but not in neutrophils, which have lower levels of CLRs. Indeed, multiple CLR-deficient mice failed to control *C. albicans* growth due to an inadequate inflammatory response from monocytes, and induced hyper-inflammatory responses leading to organ failure. Thus, multiple CLRs cooperate in removing *C. albicans* and preserving organ function during infection.

*C. albicans* triggered pyroptosis of macrophages by caspase-1, an apoptosis-associated speck-like protein containing a caspase recruitment domain (ASC), and the NLR family pyrin domain, containing 3 (NLRP3) inflammasome. Pyroptosis was dependent on the transcription factor sterol uptake control protein 2 (*UPC2)* in *C. albicans*, which has a role in regulating ergosterol production and resistance to antifungal agents [34]. *C. albicans* hyphae formation triggered the NLRP3 inflammasome in macrophages [35], especially hyphae-derived candidalysin-induced NLRP3 inflammasome-mediated pyroptosis and the IL-1β secretion for macrophages and mononuclear phagocytes [36,37]. *C. albicans* secreted aspartic proteases 2 and 6, activated the NLRP3 inflammasome, and induced IL-1β, TNF-α, and IL-6 production in monocytes, macrophages, and dendritic cells [38]. β-glucans also triggered NLRP3 inflammasome-dependent IL-1β secretion in immune cells, including dendritic cells, through complement receptor 3 and dectin-1 signaling [39]. NLRP3-deficiency affected the control of *C. albicans* infections, induction of T helper (Th)17 cell responses, and production of proinflammatory cytokines in macrophages and dendritic cells, while NLRP10-deficiency reduced Th1 and Th17 responses without affecting the production of innate proinflammatory cytokines [40]. Thus, NLRP3 may have a key role in the innate immune responses against *C. albicans*, whereas NLRP10 is important for anti-fungal adaptive immunity.

*C. albicans* can inhibit nitric oxide production in human macrophages by enhancing host arginase activity [41]. Purified chitin, or the increased exposure of chitin, induced arginase activity in host cells, whereas an arginase inhibitor or chitinase inhibitor recovered nitric oxide production and the capacity for killing *C. albicans*. However, *C. albicans* affects macrophage activation and modulates arginase metabolism to avoid death.

*C. albicans* is also internalized by dendritic cells, which are specialized antigen-presenting cells found in the dental pulp and periapical lesions [42,43]. The yeast form enters via mannose receptors that induce proinflammatory cytokines and are killed after internalization. However, hyphal forms that enter through complement receptor 3 are found in the cytoplasm of dendritic cells [43,44].

Mast cells are long-lived immune cells found in the pulp and periapical lesions that produce TNF-α, IL-6, IL-13, and IL-4 in response to *C. albicans* [45]. They engulf *C. albicans* hyphae via α-tubulin cytoskeletal rearrangement and the accumulation of the late phase marker lysosomal-associated membrane protein-1 (LAMP1) vesicles at the phagocytic synapses [45]. Mast cells infected by *C. albicans* enhanced chemotaxis and the movement of macrophages to the infection site, whereas uninfected mast cells inhibited the macrophage phagocytosis of *C. albicans*. Thus, mast cells inhibited *C. albicans* growth and regulated macrophage responses.

IL-17 was released by T cell receptor (TCR) αβ^+^ cells when they were stimulated with *C. albicans*. A *C. albicans* mutant that was unable to form hyphae was impaired in inducing TCRαβ^+^ cell proliferation and IL-17a expression. Also, candidalysin-deficient *C. albicans* lost the ability to induce IL-17a production and innate TCRαβ^+^ cell proliferation. Thus, candidalysin in *C. albicans* hyphae may have a key role in inducing the inflammatory response in lymphocytes [46].

### 2.2. Recognition of C. albicans by Non-Immune Cells 

There are also non-immune cells in pulp and periapical lesions, including pulp cells, periodontal ligament (PDL) cells, fibroblasts, and epithelial cells, that respond to *C. albicans* (Table 2). They respond through other receptors, including specialized PRRs such as TLR, CLR, NLR, and RLR.

Oral epithelial cells have PRRs that mediate immune responses. Ephrin type A receptor 2 (EphA2) on these cells bound β-glucans on *C. albicans*, which induced proinflammatory and antifungal responses via the activation of the signal transducer and the activator of transcription 3 (Stat3), and via mitogen-activated protein kinase (MAPK) signaling [47]. EphA2-deficient mice demonstrated reduced inflammatory responses and IL-17 signaling, resulting in severe disease. Thus, EphA2 PRR on oral epithelial cells is a key to sensing *C. albicans* and inducing an immune response.

The oral epithelium responded to β-glucan of *C. albicans* with elevated heme oxygenase-1 (HO-1) expression via the generation of intracellular reactive oxygen species, p38 MAPK phosphorylation, and nuclear factor erythroid 2–related factor 2 (Nrf2) translocation into the nuclei [48]. HO-1 overexpression led to reduced cytotoxicity and injury caused by oxidative stress [49]. Thus, the oral epithelial cell expression of HO-1 in response to *C. albicans* β-glucan may be an important protective mechanism. The Als3p and Ssa1p genes of *C. albicans* also triggered local immune responses by inducing the production of cytokines and chemokines in oral epithelial cells [50]. *C. albicans* up-regulated the secretion of galectin-3 on human gingival epithelial cells and fibroblasts via cytoskeletal changes, protease activity, or phosphoinositide 3-kinases (PI3K) signaling [51]. 

The oral epithelial cell responses to *C. albicans* were different from those of vaginal epithelial cells [50]. Oral epithelial cells infected by *C. albicans* are efficient at releasing cytokines such as IL-1α or IL-1β, and chemokines such as IL-8 or MIP-3 α, rather than directly killing *C. albicans*. Conversely, *C. albicans* can kill oral epithelial cells. They interact with oral epithelial cells, invade them, and rapidly activate caspase-dependent apoptosis, which results in cell death [52]. However, *C. albicans*-infected oral epithelial cells can enhance *C. albicans* hyphal damage by neutrophils [53]. The supernatant of *C. albicans*-infected oral epithelial cells stimulated neutrophils to suppress the metabolic activity of hyphal *C. albicans.* This was partially inhibited by anti-IL-1α and IL-1 receptor neutralizing antibodies. Thus, *C. albicans*-infected oral epithelial cells can stimulate neutrophil anti-hyphal activity though cytokines.

*C. albicans* can regulate the production of chemokines, such as fractalkine/C-X_3_-C Motif Chemokine Ligand 1 (CX3CL1), which act as chemoattractants and adhesion molecules in fibroblasts [54]. *C. albicans* increased the mRNA levels of several chemokines except CX3CL1 in keratinocytes, and they induced CX3CL1 expression in human oral fibroblasts. CX3CL1 chemokines showed antifungal effects against *C. albicans*. Thus, CX3CL1 from oral fibroblasts may play a key role in the oral immune response to *C. albicans* infection.

PDL cells are also involved in immune responses when they encounter pathogenic microorganisms. *C. albicans* in biofilms persistently stimulated PDL cells expression of IL-6 and TNF-α, or IL-1β and the receptor activator of nuclear factor kappa-Β ligand (RANKL), which are involved in bone resorption [55]. Additionally, *C. albicans* reduced PDL cells expression of IL-10, which is an anti-inflammatory cytokine that inhibits the expression of proinflammatory cytokines.

Dental pulp cells are involved in the repair and regeneration of pulpal tissues and dentin. However, they can be killed by *C. albicans* [56]. *C. albicans* hyphae invade pulp cells and secrete degradative proteases that cause cell damage. *C. albicans* enhanced pulp cell expression of the C-type lectin domain containing 7A (CLEC7A), TLR2, and TLR4, which act as fungus-associated receptors and produce proinflammatory cytokines such as interferon (IFN)-α. *C. albicans* infection also increased the production of FOXO3a, which is a key transcription factor involved in the immune response. Thus, dental pulp cells may influence the production of proinflammatory cytokines against *C. albicans.*

All of these cells may have other receptors involved in recognizing fungi. Additionally, other cell types such as odontoblasts may interact and respond to *C. albicans* and have a role in the immune response to *C. albicans* infection. Therefore, further studies are needed to understand the complex immune response against *C. albicans* in pulp and periapical tissues.

### 2.3. Myeloid Cells Affected by C. albicans Infection

In bone marrow, myeloid cells that originate from hematopoietic stem cells give rise to osteoclasts, macrophages, granulocytes, and dendritic cells. Siglec-15 is a member of the glycan-recognition proteins that are primarily expressed on myeloid cells. Siglec-15 is a risk allele for recurrent vulvovaginal infection by *C. albicans* [57] (Table 3). Indeed, PBMCs from donors with the Siglec-15 allele increased production of T cell cytokines such as IL-17, IL-22, and interferon-γ by *C. albicans* more than those of donors without the risk allele. *C. albicans* increased Siglec-15 mRNA expression in human blood myeloid cells and epithelial cells, and directly bound Siglec-15. Osteoclast differentiation from the osteoclast precursor was reduced by using anti-Siglec-15 antibodies. Sialylated ligands induced osteoclast differentiation by the recognition of Siglec-15. Thus, *C. albicans* recognition by Siglec-15 might participate in osteoclast differentiation and fusion.

*C. albicans* can induce IL-23 production in monocytes, macrophages, dendritic cells, and neutrophils [58]. IL-23 was important for protection against *C. albicans* infection in a mouse model in which IL-23-deficient mice were more susceptible to *C. albicans* infection. IL-23 protected myeloid cells from apoptosis and promoted host defenses against systemic candidiasis [59]. However, IL-23 is associated with osteoclast differentiation in mouse macrophages and may thereby affect bone resorption during *C. albicans* infection.

Conversely, osteoclastogenesis was reported to be inhibited by dectin-1 signaling. In osteoclast precursors, the activation of dectin-1 inhibited RANKL-mediated osteoclast differentiation via the inhibition of the nuclear factor of activated T cells 1 (NFATc1), dendritic cell-specific transmembrane protein (DC-STAMP), tartrate resistant acid phosphatase (TRAP), and cathepsin K induced by IL-33 [60]. Additionally, Dectin-1 activation inhibited osteoclast-mediated bone resorption. *C. albicans* β-1-3-glucans recognition by dectin-1 may regulate osteoclastogenesis. The recognition of *C. albicans*-derived β-glucan by dectin-1 stimulated nociceptors to induce the production of calcitonin gene-related peptides (CGRP) [61]. The CGRP inhibited β-glucan-induced inflammation and multinucleation of osteoclasts via the inhibition of NF-κB p65 and the inhibition of actin polymerization, respectively. Thus, Dectin-1 signaling by β-glucan may regulate bone destruction via the induction of CGRP. Experimental osteoarthritis induced by *C. albicans* in rats showed irregular new bone formation and resorptions, leading to severe deformation of the joint bones [62,63]. However, further study is needed to determine the effects of *C. albicans* on oral bone turnover in periapical lesions.

## 3. Treatment Options for Endodontic *C. albicans* Infection

### 3.1. Intraradicular Eradication

#### 3.1.1. Mechanical Instrumentation

Most intracanal pathogens could be removed during the root canal treatment procedure, including pulp extirpation, root canal enlargement and preparation, and intracanal disinfection techniques. However, *C. albicans* biofilm and the smear layer on root canal walls resist and survive treatment. Although the outer layer of microbial biofilm is directly affected, the extracellular matrix structure prevents treatment procedures from affecting deeper layers of viable microorganisms. Also, microbes remain sequestered within complex root canal spaces and inside dentinal tubules as endodontic instruments cannot reach all intracanal surfaces. Such intratubular microorganisms are the primary cause of microbial resistance to treatment, and post-treatment recurrence of apical periodontitis.

#### 3.1.2. Chemical Irrigants

##### Sodium Hypochlorite

The most widely used endodontic irrigant is sodium hypochlorite (NaOCl), which has broad spectrum antimicrobial activity and necrotic tissue dissolving properties. Its antimicrobial actions are through free chlorine release, which is pH-dependent and oxidizes amino acid sulfhydryl groups [64] to the di-sulfide form (S-S), causing protein breakdown [65]. Fungicidal actions involve the denaturation of proteins and the inhibition of enzymatic reactions, which reduce cell attachment and function [66]. However, these cytotoxic properties and the tissue dissolving capacity limit NaOCl application to short-term intracanal use.

##### Chlorhexidine Digluconate

Another commonly used endodontic irrigant is chlorhexidine digluconate (CHX), which consists of cationic molecules that bind dentin surfaces to provide prolonged resistance to microbial colonization (substantivity) and broad-spectrum antimicrobial activity [67]. Cationic CHX molecules interact with negatively-charged membrane phospholipids to enter and permeabilize microbial cells [68]. CHX altered cell walls and nucleoprotein coagulation in microbes, including *C. albicans* [69,70], and CHX was reported to be more effective against *C. albicans* compared with calcium hydroxide [71]. Additionally, CHX binds to hydroxyapatite to reduce microbial colonization on dentin surfaces, exerting substantive activity [67]. CHX showed superior antifungal activity compared with calcium hydroxide up to a 400 µm depth of dentinal tubules in a human dentin block model [69]. However, dentin components (hydroxyapatite, dentin matrix, and type I collagen), microbial cells, and inflammatory exudates within infected root canals may potentially negate CHX’s effects [72]. Other limitations include an incapacity to disrupt biofilms [70], remove smear layers [69], or dissolve tissues [73]. Therefore, CHX must be accompanied by other irrigants or medicaments and endodontic instrumentation. Unfortunately, CHX used in conjunction with NaOCl risks the formation of para-chloroaniline, which is a toxic aromatic amine that may cause cyanosis.

##### Alexidine Digluconate

Alexidine digluconate (AXD) is an anticancer drug that targets a mitochondrial tyrosine phosphatase (PTPMT1) and causes mitochondrial apoptosis [64]. AXD inhibited planktonic growth and biofilm formation of diverse fungi and showed fungicidal effects [74]. ALX demonstrated longer antimicrobial substantivity than CHX [75], and there were no precipitates or toxic byproducts when combined with NaOCl [76]. Thus, ALX has recently been in the spotlight as a potential antibiofilm and antifungal agent for diverse fungal infections and for applications in endodontics.

##### Ethylene Diamine Tetraacetic Acid 

In endodontics, ethylene diamine tetraacetic acid (EDTA) is not used as an antimicrobial, but to remove the smear layer from root canal walls. This exposes the dentin surface and dentinal tubule orifices and increases the antimicrobial effects of other agents [77]. The antifungal properties of EDTA were first studied by Sen et al. [78], and there are two mechanisms for its antifungal activity in preventing colonization and inhibiting growth. First, EDTA chelates calcium ions (Ca^2+^) and inhibits *C. albicans* binding to proteins, which interferes with adherence and colonization. Secondly, EDTA can remove Ca^2+^ from cell walls to make them collapse and inhibit enzyme reactions [79] and other divalent ions (Mg^2+^, Mn^2+^ and Zn^2+^) to prevent the growth and morphogenesis of *C*. a*lbicans* [80,81,82]. Therefore, copious irrigation with EDTA during root canal disinfection is particularly important in persistent cases or for medically compromised patients who are susceptible to oral candidiasis.

#### 3.1.3. Intracanal Medicaments

##### Calcium Hydroxide

Calcium hydroxide is the most widely used intracanal medicament in endodontics. The hydroxyl ions released from calcium hydroxide react intensively with several biomolecules. The reaction is nonspecific and these highly oxidant free radicals gather at their sites of generation. Hydroxyl ions damage bacterial cytoplasmic membranes, denature proteins, and damage DNA, which is fatal for bacterial cells. The hydroxyl ions alkaline pH gradient damages cytoplasmic membrane proteins and alters membrane integrity. They act on diverse aspects, including organic components, nutrient transport, and the phospholipids or unsaturated fatty acids of cellular membranes. However, *C. albicans* survives at a wide range of pH values (3.0–8.0) and is resistant to calcium hydroxide [83], which has demonstrated little or no effect on intracanal fungal infections [84]. Extracellular pH controls *C. albicans* dimorphism, and thereby its pathogenicity. In alkaline pH, *C. albicans* adaptations involve filamentous growth, which is essential for pathogenicity [85]. Like other fungi, *C. albicans* mutants that lack pH-response transcription factor PacC for pH regulation are defective in filamentous growth [86,87]. Additionally, *C. albicans* can utilize the Ca^2+^ released from calcium hydroxide as an essential for growth [84].

##### Antimicrobial Peptides 

The intracanal application of antibiotics was common for endodontic treatment in the 1950s and 1960s. Most antibiotic pastes contained an antifungal component, usually nystatin or sodium caprylate. However, since the 1970s, intracanal antibiotics have been less frequently used due to risks of microbial resistance and host sensitization. Commonly used antibacterial irrigants and medications with limited antifungal effects may favor the overgrowth of yeasts within root canals [88]. Until recently, the importance of antifungal considerations in endodontic therapy received little attention.

Antimicrobial peptides (AMPs) that act through cell lysis could be promising as antifungal and antibiofilm intracanal medicaments. They are amphipathic and both neutral and positively charged molecules that bind and disrupt cellular membranes [89]. They traverse cellular membranes and interact with certain molecules or interfere with the cell wall or the synthesis of essential components, such as glucan and chitin [90]. These include defensins, which are mammalian peptides present in numerous organisms. The unique β-sheet structures of defensins stabilized by three disulfide bonds form highly amphipathic molecules with variably cationic charges. The three-dimensional folds enable reduced molecular size, whereas other antimicrobial peptides form helical structures. Their cationic properties make them electrostatically bond to membranes and form multimeric pores, which leak essential minerals and metabolites from microbial cells [91].

**Figure 3:**
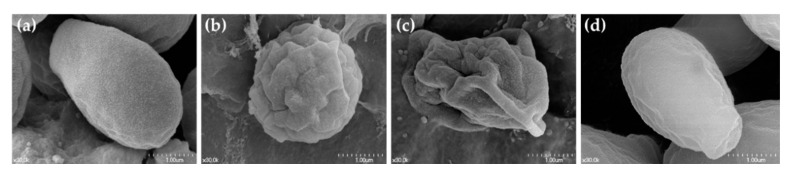
Scanning electron microscopic view of *Candida albicans* cell membranes disrupted by endodontic irrigants/medications (30,000×). (**a**) Saline (control), (**b**) chlorhexidine gel, (**c**) synthetic HBD3-C15, and (**d**) calcium hydroxide.

In endodontics, human beta defensin-3 (HBD3) was investigated for antibacterial and antifungal effects. Synthetic HBD3-C15 has fungicidal effects that may involve multifarious mechanisms. Like CHX, the antimicrobial action of HBD3 derivatives involves membrane permeabilization [69,92,93,94,95,96,97,98]. Their cationic amino acid residues and capacity to form dimers contribute to the depolarization and disruption of negatively charged microbial cell membrane lipopolysaccharides and lipoteichoic acid. HBD3 derivatives displace the Ca^2+^ or Mg^2+^ ions that normally bridge lipopolysaccharide molecules to form a pore complex on the membrane, and thereby depolarize and lyze bacterial cells (Figure 3) [98]. For enhanced clinical applications, these AMPs can be dissolved in various vehicles (aqueous, gel, etc.) and administered through an injectable syringe.

##### Antifungal Agents

Safe and effective antifungal agents are in increasing demand due to the expanding numbers of immunocompromised patients at risk of invasive fungal infections. However, the effects of antifungals, including nystatin and azole-based agents against intracanal fungal infections, are not well understood. This is largely due to deviating results from antimicrobial susceptibility tests to evaluate the antifungal capacity of agents or the resistance of strains. Waltimo et al. (100) reported differing susceptibilities to various antifungals for 70 strains of *C. albicans* isolated from cases of persistent apical periodontitis and of marginal periodontitis. They showed the limitations of in vitro studies for antifungal agents in endodontic applications. For example, propolis is a natural derivative that is well known for its antifungal activity against oral *C. albicans* infections [99], but little is known about its intracanal effects. Unlike oral manifestations, propolis antifungal effects in a *C. albicans*-infected human dentin disc model were reported to be significantly inferior than CHX [100,101].

#### 3.1.4. Root Canal Obturation

Root canal obturations aim to obtain a ‘fluid-tight seal’ of the root canal system from periradicular tissues, which starves the remaining microorganisms and prevents re-infection. However, current obturation materials are limited by their setting properties, solubility, or cytotoxicity. Therefore, newer calcium-silicate (Ca-Si) materials that undergo setting within wet environments have gained attention for use in root canal obturation. Their hydration reactions create alkaline conditions that instill antibacterial properties [102,103], and most importantly, these materials induce intratubular biomineralization. Ca-Si-based hydroxyapatite precipitates were observed within dentinal tubules after root canals were filled, and these micromechanical biomineralized structures were considered as favorable retention [104,105]. Interestingly, further study reported dead microorganisms entrapped by these biomineralized precipitates within dentinal tubules [106]. Additionally, Ca-Si-based materials provide an opportunity for developing innovative root canal obturation materials, such as antimicrobial agents incorporated into Ca-Si-based sealers, or Ca-Si materials incorporated into gutta percha.

### 3.2. Extraradicular Eradication

Infected root canals may occasionally progress on through apical foramen to develop into extraradicular infections of the external root surface and periapical tissues. These could involve abscesses, apical actinomycosis, and osteomyelitis [23,24,107]. However, there are few reports on the presence of yeast in periapical tissues [108,109]. Case reports show that the extraradicular presence of yeast appears to be primarily associated with immunocompromised patients [110,111]. Such cases of suspected extraradicular infection that persist despite comprehensive root canal treatments may require additional microsurgical endodontics to heal.

## 4. Conclusions

*C. albicans* may survive and flourish despite endodontic treatment, due to their capacity to form biofilm, invade dentinal tubules, and resist commonly used intracanal disinfectants. Their pathogenicity involves dental pulp and periradicular tissue cells, especially in immunocompromised patients. Therefore, clinicians should consider the possibility of fungal infection when dealing with cases of secondary or refractory apical periodontitis. These may require additional treatments, including intracanal medicaments such as CHX gel or HBD3, root canal obturation with Ca-Si-based sealers, or microsurgeries. Future research needs to establish biocompatible disinfection strategies that provide the most effective antibacterial and antifungal treatment for endodontic infections.

## Figures and Tables

**Table 1:** Immune cell recognition and response to *Candida albicans.*

Host Cell	Component of *C. albicans*	Receptor or Mechanisms	Increase	Decrease	Ref
macrophages	phospholipomannan	TLR2	TNF-a	/	[28]
human mononuclear cell, murine macrophages	purified mannan	/	TNF, IL-6	/	[29]
human mononuclear cells	O-linked mannosyl residues	TLR4	/	/	[29]
human mononuclear cells	N-linked mannosyl residues	mannose receptor	/	/	[29]
human peripheral blood mononuclear cells	mannan	mannose receptor	IL-17	/	[30]
human peripheral blood mononuclear cells	mannan, β-glucan	TLR2/denctin-1	mannose receptor-induced IL-17	/	[30]
human mononuclear cells	β-glucan	TLR2/denctin-1	induced cytokine production	/	[29]
macrophages	β-glucan	dectin-1	reactive oxygen, phagocytosis	/	[17]
macrophages	/	mincle	TNF-α	/	[31]
human macrophages	/	mannan-binding lectin	/	*C. albicans*-induced proinflammatory cytokines	[32]
monocyte	/	multiple CLR (Dectin-1, Dectin-2 and Mincle)	/	remove fungus	[33]
macrophage	transcription factor UPC2	caspase-1, ASC, and NLRP3 Inflammasome	pyroptosis	/	[34]
macrophage, monocuclear phagocytes	hyphae, hyphae-derived toxin candidalysin	NLRP3 Inflammasome	pyroptosis and IL-1β secretion	/	[35,36,37]
monocyte, macrophage, dendritic cells	Secreted aspartic proteases 2 and 6	NLRP3 inflammasome	induced IL-1β, TNF-α, and IL-6 production	/	[38]
dendritic cells	β-glucans	caspase-8 and NLRP3 inflammasome, Complement receptor 3 and Dectin-1 signaling	IL-1β secretion	/	[39]
macrophage and dendritic cell	/	NLRP10	Th1 and Th17 responses	/	[40]
human macrophages	purified chitin or increased exposure of chitin	enhancement of host arginase activity	/	nitric oxide production	[41]
dendritic cells	morphology of *C. albicans* and type of receptor mediated the entry into cells	mannose receptor/CR3	production of proinflammatory cytokines	/	[42]
mast cell	/	α-tubulin cytoskeleton rearrangement and accumulation LAMP1^+^ vesicles	production of TNF-α, IL-6, IL-13, and IL-4, phagocytosis of hyphae form of *C. albicans*, enhanced chemotaxis and movement of macrophages.	/	[45]
TCRαβ^+^ cells	hyphae, candidalysin	/	IL-17^+^ TCRαβ^+^ cell proliferation	/	[46]

**Table 2:** Recognition of *Candida albicans* by non-immune cells.

Host Cell	Component of *C. albicans*	Receptor or Mechanisms	Increase	Decrease	Ref
oral epithelium	β-glucans -containing particles	reactive oxygen species (ROS)/p38 MAPK/Nrf2	heme oxygenase-1 (HO-1) expression	/	[48]
oral epithelial cells	Als3p and Ssa1p gene	/	production of cytokine and chemokine	/	[50]
human gingival epithelial cell, fibroblasts	/	cytoskeletal changes, protease activity, or PI3K signaling	secretion of galectin-3	/	[51]
oral epithelial cells	/	/	release of cytokines such as IL-1α or IL-1β and chemokines such as IL-8 or Macrophage Inflammatory Protein (MIP)-3 α	/	[50]
oral epithelial cells	/	/	caspase-dependent apoptosis	/	[52]
oral epithelial cells	/	neutrophils, IL-1 signaling	*C. albicans* hyphae damage	*/*	[53]
oral fibroblasts	/	/	fractalkine/CX3CL1 (CX3CL1)	/	[54]
keratinocyte	/	/	increased mRNA levels of several chemokines excepting CX3CL1	/	[54]
periodontal ligament (PDL) cell	/	/	induced expression of IL-6 and TNF-α, or IL-1β, RANKL, IL-23 p19, and IL-17R	reduced IL-10 expression	[55]
dental pulp cells	Hyphal	/	cell damage, expression of CLEC7A, TLR2, and TLR4, IFN-α	/	[56]

**Table 3:** Myeloid cells affected by *Candida albicans* infection.

Host	Component of *C. albicans*	Receptor or Mechanisms	Increase	Decrease	Ref
human myeloid cells, epithelial cells	/	/	Siglec-15 messenger RNA (mRNA) expression (associated with osteoclast differentiation on osteoclast precursor)	/	[57]
monocytes, macrophages, dendritic cells, neutrophils	/	/	IL-23 (associated with osteoclast differentiation in mouse macrophage)	/	[58,59]
osteoclast precursor	/	dectin-1 signaling (β-1-3-glucans of *C. albicans* are ligands of Dectin-1), IL-33	RANKL-mediated osteoclast differentiation	/	[60]
mice	*C. albicans*-derived β-glucan	dectin-1, nociceptors	the production of calcitonin gene-related peptides	inhibit osteoporosis and osteomyelitis in response to β-glucan	[61]
rat	/	/	irregular new bone formation and resorptions, severe deformation of joint bones	/	[62,63]
